# Comparison of clinical features between patients with and without renal involvement in IgG4-related disease

**DOI:** 10.1007/s10238-025-01612-3

**Published:** 2025-03-25

**Authors:** Qixing Yu, Ganyuan He, Zhi Zhao, Jiayi Chen, Qiqi Huang, Wenke Hao, Xiangbin Mi, Wenxue Hu

**Affiliations:** 1 Department of Nephrology, Guangdong Provincial People’s Hospital (Guangdong Academy of Medical Sciences), Guangdong Provincial Geriatrics Institute, Southern Medical University, Guangzhou, China; 2 Department of Dermatology, Zhujiang Hospital, Southern Medical University, Guangzhou, Guangdong China

**Keywords:** IgG4-related disease, Kidney injury, Basophils, Disease relapse, Prediction model

## Abstract

**Objective:**

The kidney is one of the organs most frequently affected in immunoglobulin G4-related disease (IgG4-RD). Early identification of IgG4-RD with renal injury by clinical features is a current challenge. There is a paucity of data regarding the clinical features of renal involvement in IgG4-RD.

**Materials:**

Patients with the diagnosis of IgG4-RD with and without renal injury were included in the retrospective cohort study. Cox regression analyses were used to investigate the risk factors for disease relapse and to construct the nomogram model.

**Results:**

From December 2014 to February 2022, 54 patients with IgG4-RD were retrospectively enrolled. Renal involvement in IgG4-RD was observed in 55.6% of the patients. The differences of age and lacrimal gland accumulation were statistically significant (*P* < 0.001, and* P* = 0.034, respectively). Age was significantly higher in the kidney injury group. Regarding laboratory findings, basophil counts, hemoglobin levels, and serum cholinesterase level were significantly lower in patients with renal involvement (*P* = 0.033, *P* = 0.006 and *P* = 0.019). Erythrocyte sedimentation rate level was significantly higher in patients with renal involvement (*P* = 0.017). Seven (23.4%) patients in the kidney injury group relapsed during follow-up with mean recurrence time 9.86 ± 7.08 months. Early diagnosis plays a key role in patient outcomes. Female, elevated erythrocyte sedimentation rate level, and elevated complement component 4 are the risk factors for the disease relapse of IgG4-RD patients. Moreover, an effective nomogram model has been developed to predict disease relapse in patients with IgG4-RD.

## Introduction

IgG4-related disease (IgG4-RD) is a systemic disorder characterized by fibroinflammation that can affect multiple organs [1]. Its hallmark features include infiltration of IgG4-positive plasma cells, storiform fibrosis, and/or obliterative phlebitis, along with elevated serum levels of IgG4. Studies have demonstrated that IgG4-RD can affect nearly all organ systems [2], with the kidney being one of the most frequently affected organs.

Recent research conducted by Kimi Sumimoto et al. in 2022 revealed that IgG4-related kidney disease had a frequency of 5.16% and was the most common malignant disease (17.1%) associated with IgG4-RD [3]. However, compared to pancreas and biliary system involvement in IgG4-RD, the understanding of the mechanisms and progress of renal involvement remains limited. Therefore, further research is necessary to advance the understanding of the underlying pathogenesis and clinical significance of IgG4-RD in the kidney.

Renal involvement in IgG4-RD is commonly referred to as IgG4-related kidney disease (IgG4-RKD). IgG4-RKD encompasses renal parenchymal and renal pelvic lesions, which may directly or indirectly affect the kidney due to post-renal ureteral obstruction caused by retroperitoneal fibrosis (IgG4-RD RF) [4]. Despite improvements in test performance through the addition of new extrarenal organ items in the revised version, diagnosis remains challenging due to nonspecific clinical symptoms and inconspicuous imaging. Long-term outcomes of a large cohort study indicated that of 24 patients with IgG4-RKD, renal function deteriorated in five patients (20.8%), with 2 (8.3%) reaching end-stage renal disease (ESRD). Early identification and timely treatment for IgG4-RKD and IgG4-RPF are crucial to prevent the progression to ESRD [5]. The identification of prodromal symptoms and potential risk factors at an early stage is of utmost importance, research on clinical characteristics of renal involvement has been inadequate thus far. To bridge this gap, we conducted a comparative analysis of patients with and without renal involvement in IgG4-RD, focusing on exploring clinical features. Our investigation will provide a more substantiated and theoretical framework for clinical diagnosis and treatment.

## Methods

### Study population

In this study, we conducted a retrospective analysis of the clinical characteristics of 54 patients diagnosed with IgG4-related disease. The data were collected from the Guangdong Provincial People’s Hospital during the period from December 2014 to February 2022. All of these patients were classified as definite IgG4-RD according to the 2019 ACR/EULAR classification criteria [6]. The patient cohort was stratified into two distinct groups based on the presence or absence of renal involvement in IgG4-RD.

Patients with a diagnosis of renal insufficiency, proteinuria, hematuria, or typical changes on renal imaging met the criteria for the group with renal involvement in IgG4-RD (RI group), including renal insufficiency was defined as eGFR < 90 mL/min/1.73 m^2^, proteinuria was defined as 24 h-hoururine protein > 150 mg, and hematuria was defined as more than three red cells per high power field, and this finding should be confirmed on two or three separate urinalyses [7]. Meanwhile, patients with typical renal imaging changes were also enrolled in this group, which included thickening of the renal pelvis or hypodense bilateral renal areas on enhanced CT scan [8].

For all patients, we recorded clinical data, including age, white blood cell counts (WBC), hemoglobin (Hb), platelets (Plt), eosinophils, basophils, immunoglobulin G (IgG), immunoglobulin A (IgA), immunoglobulin M (IgM), serum immunoglobulin G4 (IgG4), complement component 3 (C3), complement component 4 (C4), serum cholinesterase level, neutrophil counts, lymphocyte counts, monocyte counts, RF positive, estimated sedimentation rate (ESR), c-reactive protein (CRP), serum cholinesterase level, and imaging information. All the clinical data were recorded from electronic records in hospital, and laboratory tests were performed in clinical laboratory of Guangdong Provincial People’s Hospital. The study involving human participants was approved by the Ethical Committee of Guangdong General Hospital. Written informed consent was obtained from the patients before the enrollment.

### Assessment of treatment outcomes

Clinical relapse was defined as a recurrence of symptoms and signs and/or worsening of imaging studies, with or without re-elevation of the serum IgG4 level [9]. The time of relapse was defined as the date of new onset or recurrence/exacerbation of disease based on symptoms, physical examination, laboratory, or radiology findings after improvement [10].

### Statistical analysis

All statistical analyses were performed using SPSS Statistics version 22.0 (SPSS Inc., New York, USA). The data were presented as means ± standard deviation (SD) or median (IQR). Logistic regression, Kaplan–Meier analysis, and Cox regression were performed using SPSS software to evaluate the risk factors and patient relapse. A nomogram was generated by R software to calculate the probability of relapse in IgG4-RD. The ggplot2 package in R software was used for data visualization. A *p value* < 0.05 was considered statistically significant.

## Results

### Patient and demographic details

The clinical and demographic data of the study subjects are shown in Table 1. There were 54 patients with IgG4-RD entered in the final analysis. The mean age was 59.43 ± 13.47 years. The RI group was significantly older than the group without renal involvement (non-RI group) (*P* < 0.001). In addition, the RI group in the study exhibited a statistically significant decrease in both basophil counts (*P* = 0.033), hemoglobin levels (*P* = 0.006), and serum cholinesterase level (*P* = 0.019) when compared to the non-RI group. Erythrocyte sedimentation rate level was significantly higher in the RI group (*P* = 0.017). There were no significant differences in male predominance, frequencies the number of extrarenal involved organs, the levels of serum IgG4, serum levels of IgG, total IgG minus IgG4, IgA, IgM, eosinophils counts, C3, C4, and CRP (Table 1).

**Table 1:** Clinical and laboratory features of patients at diagnosis of IgG4-RD

Characteristics at baseline	All patients (*n* = 54)	RI group (*n* = 30)	Non-RI group (*n* = 24)	*P* value
Demographic features				
Age, mean ± SD	59.43 ± 13.47	65.43 ± 9.19	51.92 ± 14.33	< 0.001***
Male, n (%)	42(77.8)	24(80.0)	18(75.0)	0.661
No. of involved extrarenal organs, mean ± SD	1.69 ± 0.89	1.69 ± 0.89	1.83 ± 0.86	0.460
Organ involvement (n%)				
Lacrimal gland	11(20.3%)	3(10.0%)	8(33.3%)	0.034*
Salivary gland	6(11.1%)	4(13.3%)	2(8.3%)	0.561
Parotid gland	5(9.3%)	1(3.3%)	4(16.7%)	0.093
Lymph node	29(53.7%)	14(46.7%)	15(62.5%)	0.246
Lung	7(13.0%)	3(10.0%)	4(16.7%)	0.469
Pancreas and bile duct	15(27.8%)	6(20.0%)	9(37.5%)	0.154
Retroperitoneum and aorta	6(11.1%)	5(16.7%)	1(4.2%)	0.146
Serological features				
WBC (10^9^/L)	7.55(6.11,9.23)	7.55(6.09,9.23)	7.70(6.08,11.37)	0.632
Hb (g/L)	118.15 ± 22.805	110.70 ± 24.67	127.46 ± 16.36	0.006**
PLT (10^9^/L)	263.00(199.75,336.50)	259.00(184.50,347.25)	274.00(204.00,332.75)	0.862
Neutrophils counts (10^9^/L)	0.612 ± 0.149	0.635 ± 0.110	0.584 ± 0.186	0.236
Lymphocyte counts (10^9^/L)	0.248 ± 0.114	0.233 ± 0.090	0.262 ± 0.138	0.257
Monocyte counts (10^9^/L)	0.082(0.066,0.096)	0.080(0.065,0.096)	0.085(0.067,0.098)	0.715
Eosinophil counts (10^9^/L)	0.145(0.068,0.448)	0.135(0.057,0.323)	0.195(0.014,0.126)	0.418
Basophil counts (10^9^/L)	0.025(0.010,0.043)	0.020(0.010,0.030)	0.040(0.020,0.068)	0.033*
ESR (mm/H)	54.50(26.75,81.25)	55.07(34.75,88.75)	42.50(12.00,55.07)	0.017*
CRP (mg/L)	6.20(1.67,38.96)	20.63(2.27,53.91)	4.96(0.81,28.65)	0.055
IgG (g/L)	20.35(15.99,29.86)	21.22(16.63,35.94)	18.79(15.01,26.60)	0.347
IgA (g/L)	2.22(1.33,3.72)	2.59(16.62,35.94)	2.32(1.40,3.54)	0.280
IgM (g/L)	0.89(0.66,1.53)	1.20(0.69,1.96)	0.92(0.52,1.55)	0.088
Serum IgG4, median (g/L)	10.41(4.45,15.20)	11.86(3.64,11.86)	10.44(4.39,16.80)	0.529
C3 (mg/L)	841.42 ± 295.50	780.42 ± 320.90	917.67 ± 245.87	0.090
C4 (mg/L)	182.15(111.25,242.25)	183.87(183.87,246.25)	182.15(91.75,212.00)	0.882
RF positive (n, %)	20.00(8.68,57.38)	20.10(8.68,81.89)	20.00(8.83,81.89)	0.551
Serum cholinesterase level (U/L)	5285.06 ± 2458.69	4542.19 ± 2202.57	6157.13 ± 25.01.69	0.019*
Physiology (n%)				
IgG +	31(57.4%)	19(63.3%)	12(50.0%)	0.325
IgG4 +	30(55.6%)	19 (63.3%)	11 (45.8%)	0.198
IgG4 + /IgG + > 40%	24(44.4%)	16(53.3%)	8(44.4%)	0.142

WBC, white blood cell; Hb, hemoglobin; Plt, platelet; ESR, erythrocyte sedimentation rate; CRP, c-reactive protein; IgG, immunoglobulin G, IgA immunoglobulin A; IgM immunoglobulin M; IgG4, serum immunoglobulin G4; C3, complements 3; C4, complements 4. The statistics were presented as mean ± SD and median (quartile)

*P < 0.05, **P < 0.01, ***P < 0.001

**Fig. 1:**
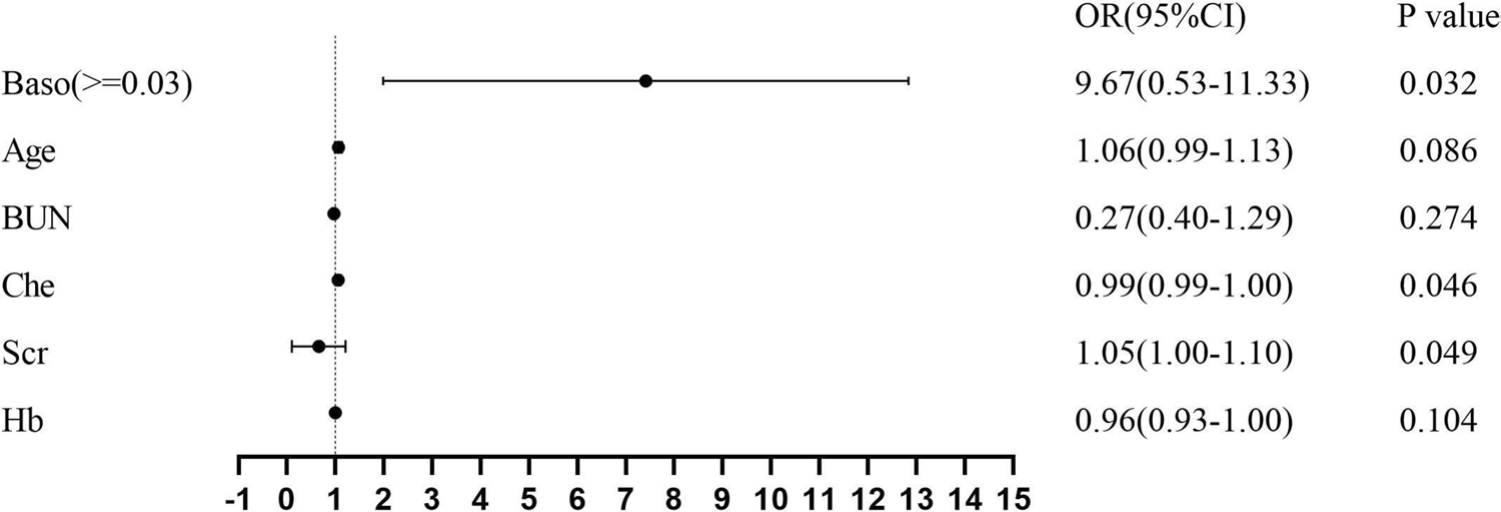
Univariate logistic regression analyses for the risk factors

We used X-tile software to determine the optimal cutoff points of basophil counts based on the outcome. And the cutoff point was 0.03*10^9^/L. In the multivariate logistic regression model, higher basophil counts (OR, 9.67; 95% CI, 0.53–11.33; *P* = 0.032) was the detrimental risk factor for the RI group (Fig. 1). As shown in Table 2, the risk of IgG4-RD patients with renal injury was higher in patients with higher basophil counts than in IgG4-RD patients without higher basophil counts in the unadjusted model (model 1: OR, 4.85; 95% CI,1.51–15.53; *P* = 0.008). After adjusting for covariates (model 3), this association remained significant (OR, 23.65; 95% CI, 2.86–195.46; *P *= 0.003) (Table 2).

**Table 2:** Association of Baso with IgG4-RD with renal injury in cross-sectional studies

Variables	Model 1			Model 2			Model 3	
	OR (95%CI)	*P*		OR (95%CI)	*P*		OR (95%CI)	*P*
Baso								
< 0.03	Ref			Ref			Ref	
> = 0.03	4.85 (1.51–15.53)	0.008		16.19 (2.61–100.52)	0.003		23.65 (2.86–195.46)	0.003

Model 1: Crude

Model 2: Adjust: Sex, age

Model 3: Adjust: Sex, age, hypertension, diabetes, kidney disease

OR: Odds ratio, CI: confidence interval

### Cumulative organs

Compared with the RI group, patients in the non-RI group showed a greater number of involved organs (1.83 ± 0.86 vs 1.69 ± 0.89,* P* = 0.460). A higher percentage of lacrimal gland involvement were observed in the non-RI group (*P* = 0.034). There were no significant differences in other cumulative organs. Among 54 patients, six patients had retroperitoneum affected. Five patients (90%) had varying degrees of kidney impairment, including five patients both had hydronephrosis (Table 1).

Regarding the imaging analysis of the kidneys, in the RI group, the renal ultrasound of four patients showed changes in cortical echo and unclear demarcation of the cortical medulla. Five patients had hydronephrosis, and two patients had ureteral stenosis. Renal contrast-enhanced CT in one patient showed uneven renal enhancement, with a patchy area of slightly lower signal shadow was visible. Renal contrast-enhanced CT in one patient showed bilateral renal pelvis and upper ureteral dilation, and inflammation may be possible. At six months of follow-up, contrast-enhanced CT of one of the patients showed more homogeneous enhancement in kidneys.

### Renal pathology

In the study, 39 (72.2%) patients had pathological biopsies. The ratio of IgG4 + /IgG + was higher in the RI group in pathological biopsies (*P* = 0.142) (Table 1). Only one patient underwent renal biopsy in this study. Combined light microscopy, immunofluorescence, and electron microscopy confirmed a pathological diagnosis of type II membranous nephropathy with renal tubulointerstitial injury dominated by plasma cell infiltration. In addition, the patient was diagnosed with systemic lupus erythematosus (SLE) based on proteinuria, positive anti-cardiolipin, isolated C3 hypocomplementemia, and hemolytic anemia. Considering his clinical features, he was diagnosed with IgG4-associated nephropathy with lupus nephritis.

### Treatment efficacy in IgG4-RD with and without renal involvement

Twenty-six (86.7%) patients in the RI group were treated with glucocorticoids (GCs) or GCs combined with immunosuppressant agents (GCs plus IM). Seventeen (56.7%) patients received GC monotherapy. Others were treated with GCs plus IM, including cyclophosphamide (CYC) (*n* = 8, 26.7%) and leflunomide (*n* = 1, 3.4%). Eleven (36.7%) patients had remission of kidney injury from the previous at a six-month follow-up. Seven (23.4%) patients relapsed during follow-up with mean recurrence time 9.86 ± 7.08 months.

**Fig. 2:**
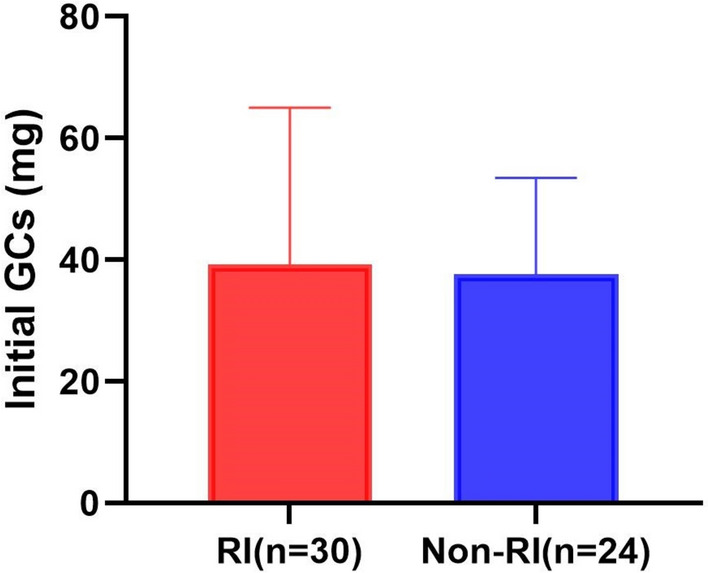
Comparison of initial GCs dose between RI group and non-RI group (*P* = 0.856)

**Fig. 3:**
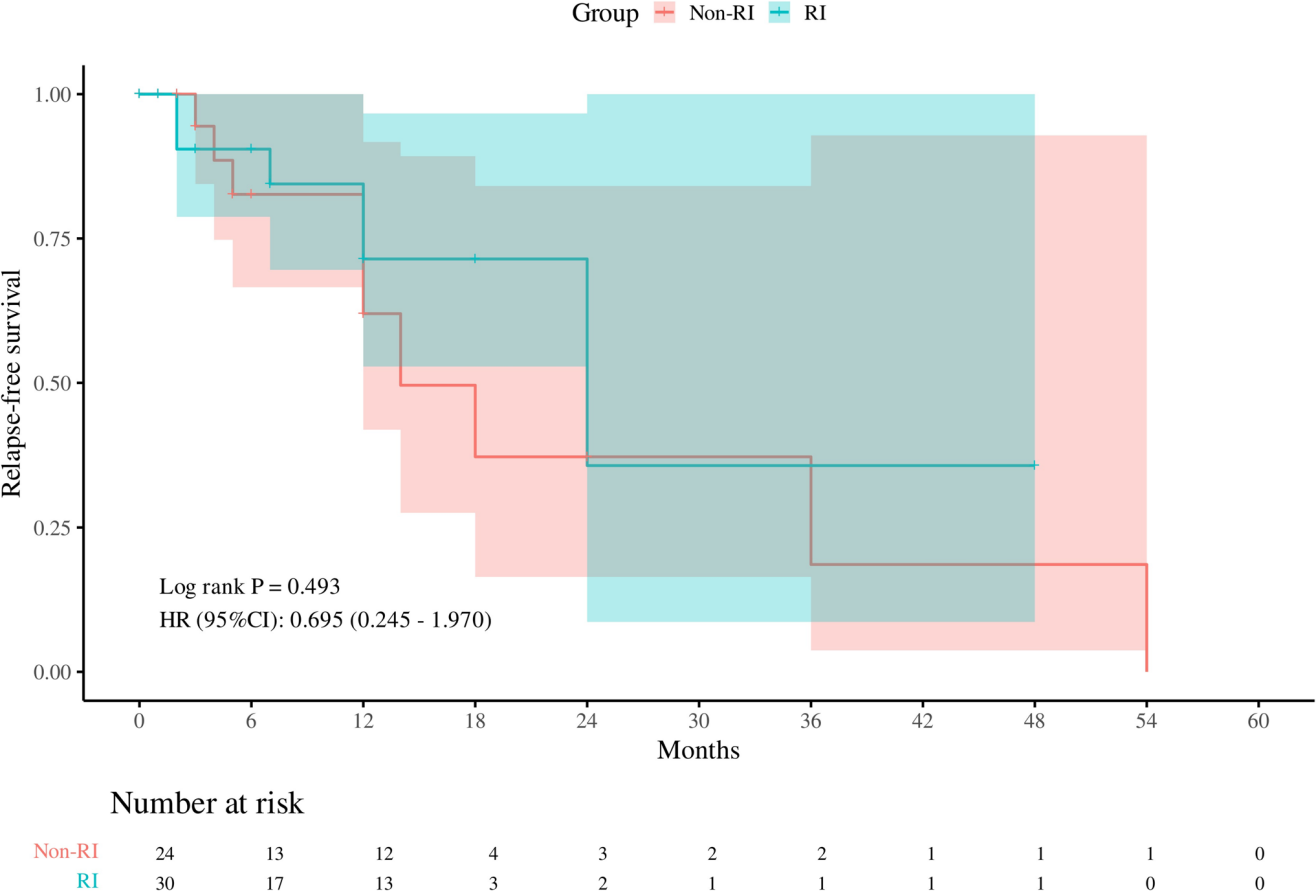
Kaplan–Meier survival analysis suggested no significant difference of relapse-free survival between two groups (*P* = 0.493)

**Fig. 4:**
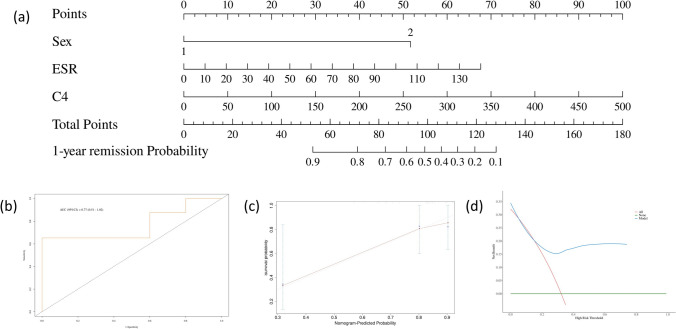
**a** A constructed nomogram for disease relapse prediction of IgG4-RD patients; **b** one-year time-dependent ROC of the model; **c** calibration curves of one-year relapse for IgG4-RD patients; **d** the decision curve of the nomogram for the relapse prediction of IgG4-RD patients

We compared the initial treatment strategies between the two groups. The initial doses of GCs in the RI group were higher than the non-RI group (38.72 ± 25.67 vs 37.63 ± 12.90, *P* = 0.856) (Fig. 2). GCs-based therapies (GCs alone or in combination with IM/RTX) were used lower frequently in with renal involvement group (86.7% vs 91.7%). The relapse rate was observed in 3.4%, 10.0%, 3.4%, and 3.4% of the patients in the RI group at 3, 6, 12, and 24 months after therapy, respectively. Kaplan–Meier survival analysis showed no significant difference in relapse-free survival between two groups (*P* = 0.493) (Fig. 3). We also attempted to identify risk factors for relapse and construct the predictive model using Cox regression and nomogram model. Through univariate Cox regression analyses (Table 3), we found that lower basophil counts (0.23 [0.06–0.9], *P* = 0.038), elevated ESR level (1.02 [1.01–1.03], *P* = 0.023), and elevated eosinophils counts (1.77 [1.14–2.77], *P* = 0.011) were significant. Further multivariate COX regression analysis revealed that female (8.19 [1.43–47.12], *P* = 0.018), elevated ESR level (1.02 [1.01–1.03], *P* = 0.013), and elevated complement component 4 (1.01[1.01–1.02], *P* = 0.037) were risk factors of disease relapse for IgG4-RD patients. Using the risk factors, we identified above, we constructed a nomogram model to predict disease relapse in IgG4-RD patients (Fig. 4a). The *C*-index value of our nomogram model was 0.79 (95% confidence interval [95% CI] = 0.79–0.97, Fig. 4b). The calibration curve in 1000 bootstrap replications displayed the comparison of predicted and observed 1-year-relapse probability (Fig. 4c). The decision curve analyses showed that our nomogram could provide a positive net benefit for the one-year disease relapse of IgG4-RD patients (Fig. 4d).

**Table 3:** Cox regression model for long-term patient survival

Factors	Univariate analysis				Multivariate analysis		
	HR	95%CI	*P* value		HR	95%CI	*P* value
Sex							
Male	Ref				Ref		
Female	2.26	0.65–7.82	0.198		8.19	1.43–47.12	0.018*
Organ number							
< 3 organs	Ref						
> = 3 organs	1.07	0.13–8.60	0.950				
Baso, 10^9^/L							
> = 0.03	Ref						
< 0.03	0.23	0.06–0.92	0.038*				
Lung	1.03	0.21–5.00	0.967				
Retroperitoneal tissue	1.07	0.13–8.60	0.950				
Lymph node	1.21	0.32–4.56	0.783				
GCs Mono	0.91	0.27–3.00	0.873				
Age	1.00	0.95–1.06	0.896				
CRP	1.01	1.00–1.02	0.207				
RF positive	1.01	1.00–1.02	0.124				
ESR	1.02	1.01–1.03	0.023*		1.02	1.01–1.03	0.013*
C3	1.00	1.00–1.00	0.608				
C4	1.00	1.00–1.01	0.188		1.01	1.01–1.02	0.037*
IgG4	1.03	1.00–1.06	0.089				
IgG	1.02	1.00–1.05	0.074				
IgA	0.96	0.83–1.12	0.605				
IgM	0.89	0.61–1.28	0.521				
Hb	0.99	0.97–1.02	0.545				
WBC	1.03	0.95–1.11	0.492				
PLT	1.00	1.00–1.01	0.234				
Eos	1.77	1.14–2.77	0.011*				

*P < 0.05, **P < 0.01, ***P < 0.001

## Discussion

IgG4-RD is a relatively newly recognized immune-mediated condition characterized by chronic inflammation that can affect multiple organs in the body and has been gradually increasing in concern in recent years. However, the understanding of IgG4-RD remains limited, and the underlying causes are not yet fully understood. The rarity of the disease and the lack of comprehensive knowledge have resulted in a scarcity of large-scale epidemiological investigations. Consequently, most of the existing studies on IgG4-RD have been derived from single-center observations or isolated case reports. To improve the awareness and understanding of IgG4-RD in clinical department, we performed a comparative analysis specifically targeting patients with and without renal involvement in IgG4-RD, focusing on analyzing their clinical features.

The incidence of IgG4-RD is observed to be higher in the age group of middle-aged to older adults, despite the possibility of occurrence at any age. The highest prevalence is observed in individuals belonging to their sixth to seventh decades of life [11]. In this study, the mean age was 59.43 ± 13.47 years, suggesting that the patient cohort primarily consisted of middle-aged to older adults, aligning with previous findings. In addition, the group of patients with renal involvement was found to be significantly older than the other group. Studies by Denic A et al. indicate that nephrons decrease in number as a result of nephrosclerosis, which is associated with aging [12, 13]. Given these findings, elderly patients should be closely monitored for renal function and urinalysis.

Recently, seminal work has highlighted a possible pathogenic role of macrophages and basophils. Basophils, the least common of the circulating granulocytes, are known to be involved in allergic inflammatory reactions as well as in immune responses to parasitic infections. Basophils have been observed to accumulate in several diseases, such as autoimmune conditions, organ rejection, and hematologic malignancies. Upon activation, these cells generate Th2-type cytokines and function as antigen-presenting cells, facilitating Th2 immune responses [14]. Recently, their role in IgG4-RD has been evaluated, particularly in relation to the activation of their Toll-like receptors (TLRs) and Nod-like receptors (NLRs) [15]. Studies have shown that basophils can stimulate the production of IgG4 by B cells in response to TLR2 and four ligands, as well as the production of BAFF and IL-13 after TLR4 activation. IgG4 production is dependent on the BAFF signaling pathway. Basophils from patients with autoimmune pancreatitis (AIP) induced a more significant IgG4 production than those from healthy controls, indicating that TLR-mediated innate immunity represented by basophils may contribute to the development of IgG4-RD in a T-independent process that involves BAFF signals [16]. Additionally, activated basophils release mediators of inflammation, including histamine, leukotrienes, prostaglandins, cytokines (IL-3, IL-4, IL-6, IL-9, and IL-13) and chemokines (CCL3 and CCL4), which are inflammatory mediators that contribute to the allergic response [17]. Nevertheless, the pathway of basophil activation in IgG4-RD remains unclear. In IgG4-RD pathophysiology, systemic fibrotic inflammation is a critical characteristic of IgG4-RD. Genetic studies indicated enrichment of genes causing kidney dysfunction in PT cells [18]. The critical role of IL-34 and CXCL16 in renal fibrosis was established by previous studies [19] [20]. Previous studies showed that blocking IL-18 and IL-33 ameliorated renal fibrosis [21]. A recent report provides and additional evidence on the pathogenic role of basophils in promoting kidney fibrosis. By applying single-cell sequencing in a murine model of kidney fibrosis (KF), Katalin Susztak and colleagues report mechanistic evidence that an increase in lymphoid and myeloid cells includes basophils in UUO kidneys whereas the fraction of renal tubular epithelial cells was decreased, and basophils secreted a large amount of IL-6 in response to IL-18 and IL-33 and interacted with TH17 cells, orchestrating kidney fibrosis development. The study showed that profibrotic PT cells expressing CXCL1 were likely responsible for basophil recruitment [22]. It is interesting that, similar to the results of this study, we found that our research also revealed a significant decrease in peripheral blood basophils among individuals with renal involvement. In addition, previous study in the chronic cardiac allograft rejection model has identified basophil-derived IL-4 as a major profibrotic cytokine [12]. It can be speculated that basophils might play an important role in the mechanism of IgG4-RD. However, the specific role of basophils in IgG4-RD remains largely unexplored. Investigating their involvement in renal fibrosis is both logical and attractive.

Our results showed that the group with kidney injury had a significantly lower Hb compared to the other group, which may be associated with insufficient secretion of erythropoietin (EPO). Erythropoietin (EPO) is a glycoprotein hormone that is primarily known for its role in regulating red blood cell production. However, it has also been found to have a protective effect on the kidneys, particularly in the context of kidney fibrosis [23]. Animal studies have demonstrated that EPO can regulate the accumulation of fibrocytes and reduce collagen production in human fibrocytes subjected to TGF-β simulation, a cytokine that induces fibrosis by activating fibroblasts [24]. For example, TGF-β1 and CTGF are key mediators of fibrosis in the kidney. A study has demonstrated that EPO exhibited a capacity to attenuate cardiac dysfunction by inhibiting interstitial fibrosis in diabetic rats. In addition, EPO has been shown to reduce inflammation in the kidney by inhibiting the production of pro-inflammatory cytokines and promoting the expression of anti-inflammatory cytokines, including the regulation of regulatory T cells (Tregs) [25]. Another study found that treatment with EPO led to an increase in the number of Tregs in the spleen and lymph nodes of mice with experimental autoimmune encephalomyelitis (EAE) [26]. Tregs may play a protective role in IgG4-RD. Some studies have found that Tregs are enriched in the affected tissues of patients with IgG4-RD, and that the number of Tregs in these tissues correlates with disease activity[27]. Overall, EPO appears to have a multi-faceted protective effect on the kidneys, which can help to prevent the development of fibrosis and promote tissue repair. Further research is required to elucidate the connection between kidney fibrosis and EPO signaling. Additionally, it is crucial to recognize the decline in Hb in patients with IgG4-RD, as it potentially contributes to organ fibrosis.

A lower level of serum cholinesterase was observed patient in without renal involvement compared to those with renal involvement in this study. In this study, serum cholinesterase level was not the detrimental risk factor for the RI group in the multivariate logistic regression model. But in Kazu Hamada studies, which showed that serum cholinesterase could be new biomarker and predicts the extent of organ involvement in IgG4-related disease [28]. Serum cholinesterase comprises acetylcholinesterase and butyrylcholinesterase (BChE). In Japan, the measurement of serum cholinesterase generally means that of BChE. The tissues with the highest BChE activity were found to be the liver, lungs, spleen, kidney, pancreas, stomach, small intestine, cerebellum, heart, and plasma [29]. The serum cholinesterase level might be a new biomarker and clinically useful predictor.

We also compared treatment outcomes between two groups. Within six months posttreatment, the kidney damage significantly ameliorated compared to the initial condition. However, most of patient still could not return to normal condition because of some irreversible damage. Subclinical disease in some patients with IgG4-RD can result in severe and irreversible damage to the affected organ, thus urgent treatment is recommended for patients with IgG4-TIN to avoid such complications [30]. The highest relapse rate occurred during drug tapering within the first six months of long-term follow-up. This aligns with existing research indicating IgG4-RD’s propensity to relapse during or after glucocorticoid tapers, particularly in cases characterized by elevated serum IgG4 levels at baseline, multi-organ involvement, and a history of disease relapse. Through the univariate and multivariate Cox regression analyses, female, elevated erythrocyte sedimentation rate level, and complement component 4 are the risk factors for the disease relapse of IgG4-RD patients. Using these risk factors of relapse, we constructed a nomogram model to predict disease relapse of IgG4-RD patients. We are calling for the use of our nomogram to help identify the subtle possibility of IgG4-RD propensity to relapse. At the same time, we have joined hands with a multidisciplinary team to call on clinicians to raise awareness and collect more clinical information and prognosis to validate our nomogram, in order to ensure their wider applicability and reliability.

Our study has the following limitation. Firstly, the statistical collection of renal biopsy samples presented challenges, which limited our understanding of the role of basophils in patients with renal involvement in IgG4-RD. Second, there are insufficient data regarding the involvement of basophils and serum cholinesterase in the development of IgG4-RD. The total number of IgG4-RD patients was still limited. Therefore, we were unable to obtain a validation cohort to verify the model we built, which we hope to complete in the subsequent research. The focus of our study was on the clinical features and serological features. In smaller sample sizes, the participants in this study may not fully represent the diversity of the broader population, and the outcomes could be more sensitive to chance. The number of cases included is relatively small at present, and we plan to increase the sample size in future studies by multicenter cohorts.

To gain a more comprehensive understanding of the clinical features in IgG4-RD with renal involvement, a larger cohort study is required. Furthermore, the specific impact of basophil and serum cholinesterase on the clinical course and outcomes of patients affected by IgG4-RD and renal involvement remains undetermined, highlighting the necessity for further investigation.

## Conclusion

In conclusion, our results showed that patients with renal involvement exhibited older age, reduced levels of Hb, serum cholinesterase, and basophils, which suggested these indicators might be involved in the pathogenesis of renal injury in IgG4-RD and have potential roles in early identification, diagnosis, and treatment. Female, elevated erythrocyte sedimentation rate level, and complement component 4 are the risk factors for the disease relapse of IgG4-RD patients. A nomogram model could help predict disease relapse in IgG4-RD patients.

## Data Availability

No datasets were generated or analysed during the current study.

## Declarations

### Clinical trial number

Clinical trial number not applicable.
